# Environmentally Persistent Pesticides: In Vitro Effects on Human Aromatase Activity and ERα Signaling

**DOI:** 10.1021/acs.chemrestox.6c00151

**Published:** 2026-08-13

**Authors:** Francesca Armellino, Sabrina Costa, Francesco Giaretto, Michela Rossi, Gianluca Catucci, Gianfranco Gilardi, Tiziana Schilirò, Giovanna Di Nardo

**Affiliations:** † Department of Life Sciences and Systems Biology, University of Torino, 10123 Torino, Italy; ‡ Department of Sciences of Public Health and Pediatrics, University of Torino, 10126 Torino, Italy

## Abstract

The presence and persistence of pesticides represent a concern for public health due to their potential activity as endocrine disrupting chemicals (EDCs) by interfering with hormone synthesis and signaling, leading to severe effects on human well-being. This work investigates the activity of a series of pesticides detected in Italian surface and groundwater on two key molecular targets of the endocrine system: human aromatase (CYP19A1), responsible for the conversion of androgens into estrogens, and estrogen receptor α (ERα), essential for the estrogen-induced signaling pathway. Out of the eight pesticides tested, imazamox, metolachlor, and nicosulfuron were found to partially inhibit aromatase activity (up to ∼20% inhibition was observed when 10 or 50 μM was applied). UV/vis spectroscopy and isothermal titration calorimetry were used to calculate dissociation constants for imazamox and metolachlor that resulted of 18.8 ± 0.8 and 1.8 ± 0.7 μM, respectively. For imazamox, it was possible also to obtain a dose–response curve and to calculate an IC50 of 5.2 ± 0.8 μM. As for the estrogen receptor α, MELN (MCF-7 ERE-β-globin Luciferase Neo) gene reporter assay revealed that quinclorac and terbuthylazine-desethyl induced nuclear ERα-mediated transcriptional activation with a mean EC50 of 9.02 × 10^–4^ and 1.83 × 10^–5^ M, respectively. The data provide new insights into pesticide interference with key molecular target enzymes and receptors of the estrogen axis to better understand the impact they could have on human health when present as persistent environmental contaminants that can bioaccumulate.

## Graphical abstract

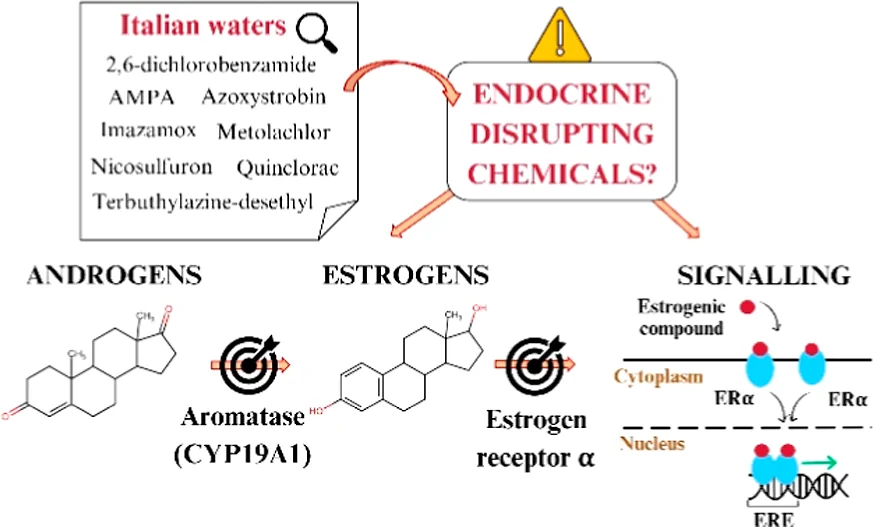

## Introduction

Endocrine disrupting chemicals (EDCs) are defined by the WHO (World Health Organization) as “exogenous substance or a mixture that alters function(s) of the endocrine system and consequently causes adverse health effects in an intact organism, its progeny, or (sub)­populations”.^1^ They include both natural compounds, such as animal and plant derivatives (e.g. phytoestrogens), and synthetic chemicals, such as pharmaceuticals, industrial solvents, phthalates, plastic additives (such as bisphenols and phthalates), heavy metals, and pesticides.^2^ The use of chemicals has become a widespread practice in modern agriculture to prevent weeds, insects, and fungal infestations and to increase agricultural productivity to guarantee food security.^3^ However, excessive use of pesticides can result in accumulation of residues in water and soil and in their entry into the food chain, thus posing a health risk factor to nontarget organisms and to consumers, who may be chronically exposed to low doses of EDCs through contaminated food and drinking water.^3–6^ Indeed, Schillirò et al. (2011)^5^ identified 34 out of 44 food samples positive for pesticides, with a maximum concentration of 6.3 μM.

Compounds 2,6-dichlorobenzamide (metabolite of dichlobenil), aminomethylphosphonic acid (AMPA, metabolite of glyphosate), azoxystrobin, imazamox, metolachlor, nicosulfuron, quinclorac, and terbuthylazine-desethyl (metabolite of terbuthylazine) were selected as candidate EDCs to be tested on two key targets of the endocrine system: aromatase and estrogen receptor α (ERα). These eight compounds were detected in Italian surface and ground waters (Table 1), according to the data collected by the Italian “Sistema Nazionale per la Protezione dell’Ambiente” (SNPA) in 2018 ^7^ and 2021.^8^ Table 1 also shows worldwide monitoring data of the eight pesticides, among which AMPA displays the highest concentrations found in Argentinian rural ground waters (2.1 μM)^10^ and in urine samples from workers in glyphosate production in China (24.6 μM).^17^

**Table 1:** Summary of Pesticides Monitoring Data in Italian Surface and Ground Water Related in 2018 and 2021 and of Pesticides Worldwide Monitoring Data, for Which the Highest Concentrations Found in the Considered Studies Are Reported

			2,6-Dichlorobenzamide	AMPA	Azoxystrobin	Imazamox	Metolachlor	Nicosulfuron	Quinclorac	Terbuthylazine-desethyl
Italian surface water	2018^a^	% detection^b^	1.9	65.8	9.5	4.0	19.5	3.9	9.1	16.0
		% > EQS^c^	0.1	52.2	1.6	1.0	3.5	0.7	4.0	0.7
	2021^d^	% detection^b^	1.0	70.2	6.3	5.3	15.1	2.0	0.7	1.1
		% > EQS^c^	0.2	45.3	1.1	1.5	1.9	0.0	0.0	0.1
Italian groundwater	2018^a^	% detection^b^	6.2	5.2	4.5	2.0	4.6	1.5	0.9	15.0
		% > EQS^c^	1.6	1.9	0.4	0.7	0.7	0.2	0.5	0.3
	2021^d^	% detection^b^	5.6	7.0	0.7	4.6	3.1	1.0	0.4	6.3
		% > EQS^c^	1.9	2.6	0.1	1.7	0.6	0.0	0.2	0.1
Worldwide	Environmental matrices^e^ [μM]	0.006 Denmark^9^	2.10 Argentina^10^	0.074 Germany^11^	0.052 Italy^12^	0.220 USA^13^	0.026 France^14^	0.256 USA^15^	0.297 (terbuthylazine) Sud Africa^16^	
	Biological matrices^f^ [μM]	N.A	24.6 China^17^	0.015 (azoxystrobin acid) USA^18^	N.A	0.599 USA^19^	N.A	N.A	0.580 Italy^20^	

a SNPA Report 334/2020.^7^

b % of water samples with at least one detection of the pesticide

c % of samples with a detected pesticide concentration over the Environmental Quality Standard for the single active compound, set at 0.1 μg/L by the Italian directive 98/83/EC.

d SNPA Report, 41/2024.^8^

e Surface and groundwater samples.

f Urine sample. N.A.: not available.

All the selected pesticides belong to the category of herbicides, except for azoxystrobin, which belongs to the fungicide family (strobilurin pesticide). Although herbicides are generally considered safe for their plant-specific action, their broad use and persistence in the environment enhance the possibility of harboring harmful effects on bacteria, fish, amphibians, and humans.^21^ In addition, strobilurins are widely exploited for fungal pathogen control due to their wide-spectrum effects and high efficacy. Their inappropriate use can impair quality and fertility of the soil by disrupting activity and abundance of soil microbiota and can exert medium–high toxic effects on aquatic ecosystems.^22,23^ Azoxystrobin, glyphosate (AMPA parental compound), imazamox, nicosulfuron, and terbuthylazine (terbuthylazine-desethyl parental compound) are currently approved in Europe, while dichlorbenil, metolachlor, and quinclorac have been banned.^24^

As many of these pesticides are classified as persistent (Table S1), the research demand heavily focuses on their impact on human and environmental health, with the necessity of studies that range from the identification of possible molecular targets and pathways to epidemiological studies. Moreover, it is crucial to elucidate dose–response relationships and mechanisms of action of such compounds.^25^ Within this research demand context, since several pesticides were found to have estrogenic and androgenic activity,^26^ this study aims to address whether the selected pesticides can bind and affect the functionality of two key targets of the endocrine system and interfere with estrogen synthesis and signaling. Aromatase (CYP19A1) is a cytochrome P450 enzyme that catalyzes the rate-limiting step in estrogen biosynthesis, converting androgens into estrogens. It is essential in many biological processes including reproductive function, brain development, bone mineralization, and adipose tissue metabolism.^27^ Once produced by aromatase, estrogens act mainly via nuclear receptors and ERα, one of the two main ER subtypes. They act as a ligand-activated transcription factor of specific target genes and control key physiological functions in reproductive, skeletal, cardiovascular, and central nervous systems, as well as in specific tissues such as breast.^28^ Therefore, any alteration of their functionality can impair reproductive and developmental processes and lead to endocrine-related cancer, as estrogens are essential for the correct growth and functioning of several tissues.^29,30^ Both proteins are well-known targets for breast cancer therapy.

Although several pesticides have been evaluated for estrogen-disrupting activity (Table S1), most available studies rely predominantly on cell-based assays that highlight their impact on aromatase gene expression or phenotypic outcomes on model systems, while direct biochemical and biophysical evidence of pesticide interaction with targets, such as human steroidogenic enzymes, remains scarce. Moreover, studies that simultaneously address interference with estrogen biosynthesis (aromatase) and estrogen receptor-mediated signaling are still limited.

To address this gap, the present work combines molecular docking, UV/vis spectroscopy, isothermal titration calorimetry, enzymatic activity assays, and ERα reporter gene analysis to provide a multilevel mechanistic characterization of pesticide interactions with two key molecular targets of the estrogen axis.

## Materials and Methods

### Materials and Chemicals

All pesticides were purchased from Sigma-Aldrich/Merck (Darmstadt, Germany). Stock solutions of the tested pesticides were prepared in potassium phosphate buffer or organic solvents (Table S2) with a final maximum solvent residue of 5.4% for UV/vis spectroscopic binding assay (quinclorac), 2.5% for activity assay (quinclorac), and 0.2% for gene reporter assay. The recombinant forms aromatase and human cytochrome P450 reductase (hCPR) were expressed in *E. coli* and purified as previously described.^31,32^

### Molecular Docking Simulations

Computational studies were conducted for the selected pesticides as a first screening for possible interaction with aromatase and ERα. Each tested pesticide was globally docked in both aromatase (PDB ID: 3EQM)^33^ and ERα (PDB ID: 1A52)^34^ after the removal of the endogenous ligands from the two structures, androstenedione (ASD) and 17β-estradiol (E2), respectively. The three-dimensional structures of the ligands were obtained from the Chemspider database (Chemspider ID: 13399; 82837; 2298772; 4025; 77711; 66024; 97278; 15359).

The analysis was carried out with the AutoDock Vina algorithm embedded in YASARA software,^35^ by using the “dock_run.mcr” macro, performing 999 docking runs with a 10 nm simulation cell built around all atoms. The YASARA macro performs structure preparation of the ligand, docking to the receptor, and results sorting by binding energy/predicted binding affinity. In YASARA, results are ranked by binding energy, where more positive energies indicate stronger binding and negative energies equate to no binding.

As a control, the analysis was repeated under the same conditions with the endogenous ligands.

The binding energies and dissociation constants were predicted by using the scoring function included in the algorithm (the higher the binding energy, the more probable the interaction).

### UV/Vis Spectroscopic Binding Assay

The assay was performed in a 1 cm cuvette with an Agilent 8453E UV/vis spectrophotometer (diode array), maintaining a constant temperature of 25 °C (Agilent 89090A Peltier Temperature Controller, Santa Clara, CA 95051, United States). Purified aromatase was diluted in a 100 mM KPi (pH 7.4), 20% v/v glycerol, and 0.1% v/v Tween-20 buffer to a final concentration of 1–2 μM in the cuvette and titrated with increasing concentrations of pesticides (5–104.7 μM for 2,6-dichlorobenzamide, 12.5–249.4 μM for AMPA and nicosulfuron, 5–95 μM for azoxystrobin and quinclorac, 14.25–138 μM for imazamox, 2–88 μM for metolachlor, and 12.4–215.3 μM for terbuthylazine-desethyl). UV/vis spectra were recorded 3 min after each pesticide addition (1 μL). Androstenedione (0.54–10.74 μM) and anastrozole (0.28–3.05 μM) were tested in the same conditions to provide a positive control for the two types of spectral shift.^31^ Since azoxystrobin was dissolved in acetonitrile, while quinclorac and terbuthylazine-desethyl were dissolved in absolute ethanol and 40% ethanol, respectively, aromatase titration with these organic solvents was performed with the same method as a control of any potential solvent interference (Figure S1). The dissociation constant (*K*_D_) of imazamox binding to aromatase was calculated by plotting the spectral transitions (ΔAbs422–ΔAbs418) as a function of imazamox concentration, fitting the data with the following equation (where L = ligand and EL = enzyme–ligand complex)

$$\left(\Delta {Abs}_{422}-\Delta {Abs}_{418}\right)=\left[{\left(\Delta {Abs}_{422}-\Delta {Abs}_{418}\right)}_{MAX}\times L_{FREE}\right]/\left(K_{D}+L_{FREE}\right)$$

$$L_{FREE}=L_{tot}-\left[EL\right]$$

$$\left[EL\right]=\left(\Delta {Abs}_{422}-\Delta {Abs}_{418}\right)\times \left[E\right]/{\left(\Delta {Abs}_{422}-\Delta {Abs}_{418}\right)}_{MAX}$$

Data are reported as means of three independent measurements, with the error bars representing the standard deviations.

### Activity Assay and Product Quantification by HPLC

To analyze the potential interference by the pesticides with the conversion of androstenedione into estrone (E1) by aromatase, activity assays were performed, followed by HPLC separation of the substrate and the product, enabling product quantification.^36^ The reaction mixtures were prepared in a 100 mM KPi (pH 7.4), 20% v/v glycerol, and 0.1% v/v Tween-20 buffer containing 0.25 μM aromatase, 0.25 μM hCPR, 0.50 μM ASD, and 500 μM NADPH. After an incubation of 10 min at 30 °C, aromatase was heat-inactivated for 10 min at 90 °C and centrifuged at 11,000*g* for 5 min. Reactions in the presence of the pesticides were carried out as described above, adding a preincubation of 5 min at 30 °C with 10 or 50 μM of each pesticide (also with 75 and 100 μM for metolachlor) to favor any possible interaction by increasing protein conformational flexibility. Reactions performed under the same conditions in the presence of 1 μM exemestane, a known aromatase inhibitor,^37^ were used as a positive control for aromatase inhibition. Negative control reactions were obtained by adding NADPH after the heat-inactivation of the protein. After centrifugation, 100 μL of the supernatant were injected into an Agilent 1260 Infinity II HPLC system connected to an Agilent ZORBAX Eclipse Plus C18 reverse phase column (4.6 × 250 mm, 5 μm). Analytes were eluted by applying an HPLC-grade acetonitrile linear gradient (25–95%, Table S3) mixed to filtered Milli-Q water, with 1 mL/min as the flow rate and 0.3 mL/min as the maximum flow gradient. Androstenedione and E1 were detected by setting the diode array detector at 237 and 280 nm, respectively. Organic solvent controls were performed by incubating the reactions with the highest percentages of solvents that correspond to the highest doses of tested pesticides (absolute ethanol for quinclorac, acetonitrile for azoxystrobin and terbuthylazine-desethyl) (Figure S2).

Aromatase activity inhibition was determined by comparing the concentration of E1 produced in the presence of pesticides to that produced in the absence of pesticides (positive control, C+). The percentage of inhibition was calculated relative to the C+ value (100%). Each reported result is the average of at least three independent experiments. In this work, the formation of intermediates (19-OH and 19-OXO ASD) was not investigated, assuming that the E1 product corresponds to 100% androstenedione conversion (in positive controls).

### ELISA Assay

A direct competitive 17β-estradiol ELISA Kit (Cloud-Clone Corp, Katy, USA) was exploited to quantify E2 produced in aromatase-catalyzed reactions in the presence of imazamox, with the aim of computing the inhibition curves of the pesticide.

Reactions were prepared in a 50 mM KPi buffer (pH 7.4), with 0.25 μM aromatase, 0.25 μM hCPR, 0.50 μM testosterone (TST), and 500 μM NADPH. After aromatase incubation (5 min at 30 °C) with increasing concentrations of imazamox (1.25–25 μM), reaction mixtures were incubated for 30 min at 30 °C and stopped by heat-inactivation for 10 min at 90 °C. Positive controls consisted in reactions in the absence of pesticides. The supernatants obtained after a centrifugation of 10 min at 11,000*g* were diluted at 1:15 in the Standard Diluent provided by the ELISA kit.

ELISA was then conducted following the manufacturer’s instructions. For each reaction, E2 concentrations were calculated according to a standard curve of known E2 concentrations (0.045–3.67 nM). Inhibition of aromatase activity was expressed as relative aromatase activity with respect to the reaction in the absence of pesticides (positive control, C+ = 100% activity), with each result being the average of three independent experiments. The IC50 value was obtained by fitting the data to a dose–response curve equation

$$y=A1+\left(A2-A1\right)/\left(1+10^{\left(LogIC50‐x\right)p}\right))$$

where A1 = bottom asymptote, A2 = top asymptote, and *p* = Hill slope.

### Isothermal Titration Calorimetry

Isothermal titration calorimetry (ITC) was performed at 25 °C in a MicroCal ITC200 instrument (Malvern Instruments, Malvern, UK). Aromatase solutions were prepared by buffer exchange with a potassium phosphate buffer (100 mM KPi, 0.1% Tween-20, pH 7.4) containing a reduced glycerol concentration (2.5% for imazamox or 10% for metolachlor), using an Amicon centrifugal filter (30 kDa MWCO).^38,39^ Ligand solutions were prepared in the flow-through of the exchanged buffer from the protein samples to ensure maximum buffer matching. The final aromatase concentration ranged from 60 to 100 μM, while the concentrations of the ligand solutions were 2.0 mM for imazamox and 1.5 mM for metolachlor. The reaction cell was filled with the aromatase sample. The pesticides were titrated into aromatase up to a pesticide/aromatase molar ratio of 9:1 (imazamox) and 3:1 (metolachlor), with the reaction cell continuously stirred at 750 rpm. The first injection (omitted in data analysis) was 0.1 μL with a 0.2 s duration for equilibrating the cell. All other injections were 1.9 μL with 8 s durations. For each experiment, a 90 s interval between injections and a reference power of 10 μW were used. Control experiments were performed by titrating both the ligand and protein solution into the buffer, maintaining the same experimental conditions. The heats of dilution obtained from the controls were subtracted from the ligand–protein titration data using OriginPro, Version 2018 (OriginLab Corporation, Northampton, MA, USA). To analyze pesticides binding properties, the integrated heat over mole of the ligand was plotted against the molar ratio (ligand to protein). By applying the one-site binding model (nonlinear regression fit, sigmoidal Boltzmann equation), the thermodynamic binding parameters were calculated.

### MELN Gene Reporter Assay

The luciferase gene reporter assay was performed using genetically modified MCF-7 cells transfected with the ERE-βGlob-Luc-SVNeo plasmid (MELN cells), kindly provided by Dr. P. Balaguer (INSERM, Montpellier, France).^40^ Cell culture conditions and assay setup followed previously published protocols,^41,42^ employing the One-Glo Luciferase Assay System (Promega, Milan, Italy). Cells were exposed for 21 h to pesticides at concentrations ranging from 3.81 × 10^–9^ to 1.00 × 10^–3^ M, except for azoxystrobin, which was tested in the range of 9.52 × 10^–10^ to 1.25 × 10^–4^ M. Pesticide cytotoxicity was assessed via WST-1 assay, and most of the tested concentrations were nontoxic. For the E2 standard curve (used as the reference compound), seven concentrations from 10^–8^ to 10^–12^ M were tested. The positive control (C+) consisted of 10^–8^ M E2, while negative controls (C−) were exposed to DMEM-F12 supplemented with 0.2% PBS or 0.2% DMSO, corresponding to the maximum concentration of solvent present in the tested conditions. Estrogenic activity induced by each treatment was expressed as relative luciferase activity, calculated as the percentage of activity induced by the treatment relative to that induced by C+ (set at 100%), while the activity of C- was set at 0%. Estrogenic activity was assessed in quadruplicate with at least two independent experiments. The relative estrogenic potency of pesticides showing a dose–response relationship was expressed as the E2 equivalency factor (EEF), calculated using the formula:

EEF = EC_50_ of E2/EC_50_ of the pesticide.

EC_50_ values, representing the concentrations that induce 50% of the maximum biological response, were calculated from dose–response curves using Probit regression, based on the relationship between relative luciferase activity and the log-transformed concentrations of each compound. To verify the involvement of nuclear estrogen receptor activation in the observed effects, cells were coexposed to each pesticide and 10^–11^ M E2 (to test for potential synergism), or to 10^–6^ M tamoxifen (*T*), an ER antagonist. Pesticides that did not show ER agonist activity were subsequently tested for potential antiestrogenic effects in two independent quadruplicate experiments. MELN cells were coexposed to 10^–11^ M E2 and either 6.25 × 10^–5^ or 3.91 × 10^–6^ M pesticide (2.44 × 10^–7^ and 3.91 × 10^–6^ M for azoxystrobin), using the same C+ and C- controls. A *T* standard curve was generated using six concentrations (10^–6^ M to 10^–11^ M), each coexposed with 10^–11^ M E2.

### Statistical Analysis

Statistical analysis of the activity assay results was performed on GraphPad Prism, Version 8.0.1 (GraphPad Software, Boston, MA, USA). A Shapiro–Wilk test followed by a two-tailed, unpaired *t*-test with Welch’s correction was used to define significant differences between the mean E1 concentrations obtained in the presence of pesticides and those obtained in the absence of pesticides. Statistical analysis of the MELN gene reporter assay results was performed using IBM SPSS Statistics 28.0 Software. A nonparametric Kruskal–Wallis test followed by the post hoc Dunnett’s test was exploited to assess significant differences compared to the negative control. Only differences with a *p*-value <0.05 were considered significant.

## Results

### Molecular Docking Analysis for Pesticides Screening

The selection of the pesticides to be investigated for a possible endocrine disrupting activity was supported not only by literature search but also by molecular docking simulations, which aimed to predict the potential binding of the pesticides to aromatase and ERα. The eight compounds were predicted to bind both aromatase and ERα, with favorable binding energy and dissociation constant values (Table 2) even if less favorable than those of physiological ligands (ASD for aromatase and E2 for ERα), with the worst predicted interaction obtained from AMPA.

**Table 2:**
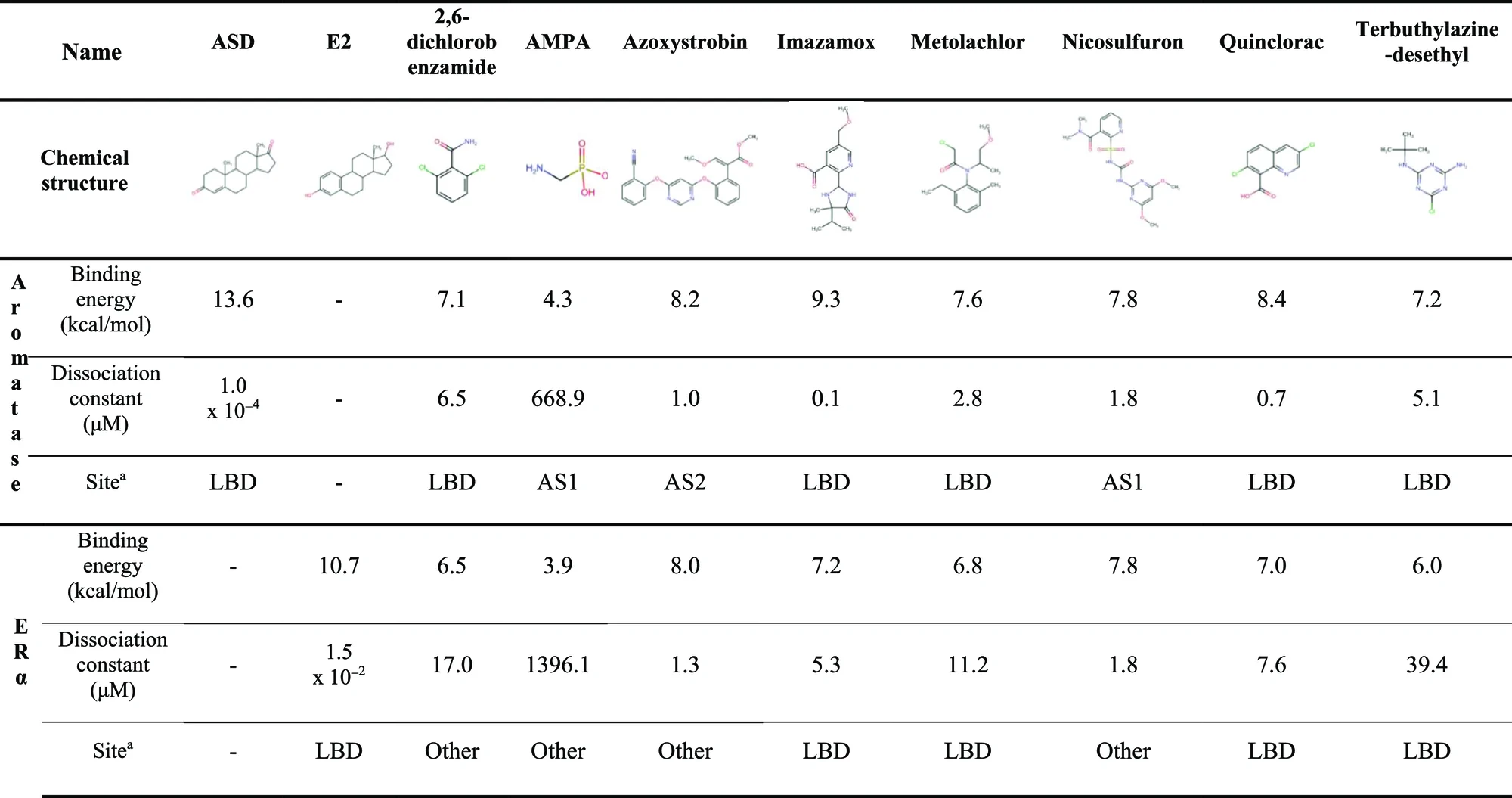
Results of Molecular Docking Simulations of Aromatase and ERα Interaction with Their Endogenous Ligands (ASD for Aromatase and E2 for ERα) and with the Eight Selected Pesticides^a^ ^b^

a LBD: ligand binding domain, AS1: allosteric site 1, and AS2: allosteric site 2.^43^

b The binding energies, dissociation constants, and docking sites are reported.

Imazamox was predicted to bind aromatase with the highest binding energy (9.3 kcal/mol) and the lowest dissociation constant (0.1 μM), followed by quinclorac (binding energy: 8.4 kcal/mol, dissociation constant: 0.7 μM), azoxystrobin (binding energy: 8.2 kcal/mol, dissociation constant: 1.0 μM), and nicosulfuron (binding energy: 7.8 kcal/mol, dissociation constant: 1.8 μM). Moreover, imazamox, metolachlor, quinclorac and terbuthylazine-desethyl were predicted to bind to the same binding site as estradiol on ERα.

It is interesting to observe that, while 5 pesticides were predicted to bind in the active site of human aromatase, 3 of them were predicted to bind in the allosteric sites identified by Sgrignani et al.^43^ and found to also bind glyphosate.^42^

Molecular docking analysis provided a useful method to predict the possible interaction of the two protein targets with pesticides, serving as a starting point for a more in-depth characterization, presented in the following results.

## Pesticides Binding to the Aromatase Active Site

In order to experimentally study whether pesticides can bind the aromatase active site, the possible interaction was assessed by UV/vis spectroscopy following the spectroscopic shift of the 417–419 nm Soret peak.^44^ A shift toward 390–394 nm (type I) is the result of a low-to-high spin transition of heme iron caused by the displacement of the distal water molecule by the ligand.^45^ In contrast, a shift to 422–426 nm (type II) is typical of nitrogen-containing chemicals that establish the sixth coordination of heme iron by direct interaction or by the mediation of a water molecule.^46,47^ For aromatase, the androstenedione substrate and the known inhibitor anastrozole are typical type I and type II (respectively) aromatase ligands.^31^ Titrations with solvents were performed as control, as described in the Materials and Methods section (Figure S1). Results showed that 2,6-dichlorobenzamide (Figure 1A), AMPA (Figure 1B), azoxystrobin (Figure 1C), nicosulfuron (Figure 1F), quinclorac (Figure 1G), and terbuthylazine-desethyl (Figure 1H) do not induce any significant spectroscopic shift, whereas the small change in the 420 nm region in the presence of quinclorac (Figure 1G, inset) could be ascribed to the solvent interference.

**Figure 1:**
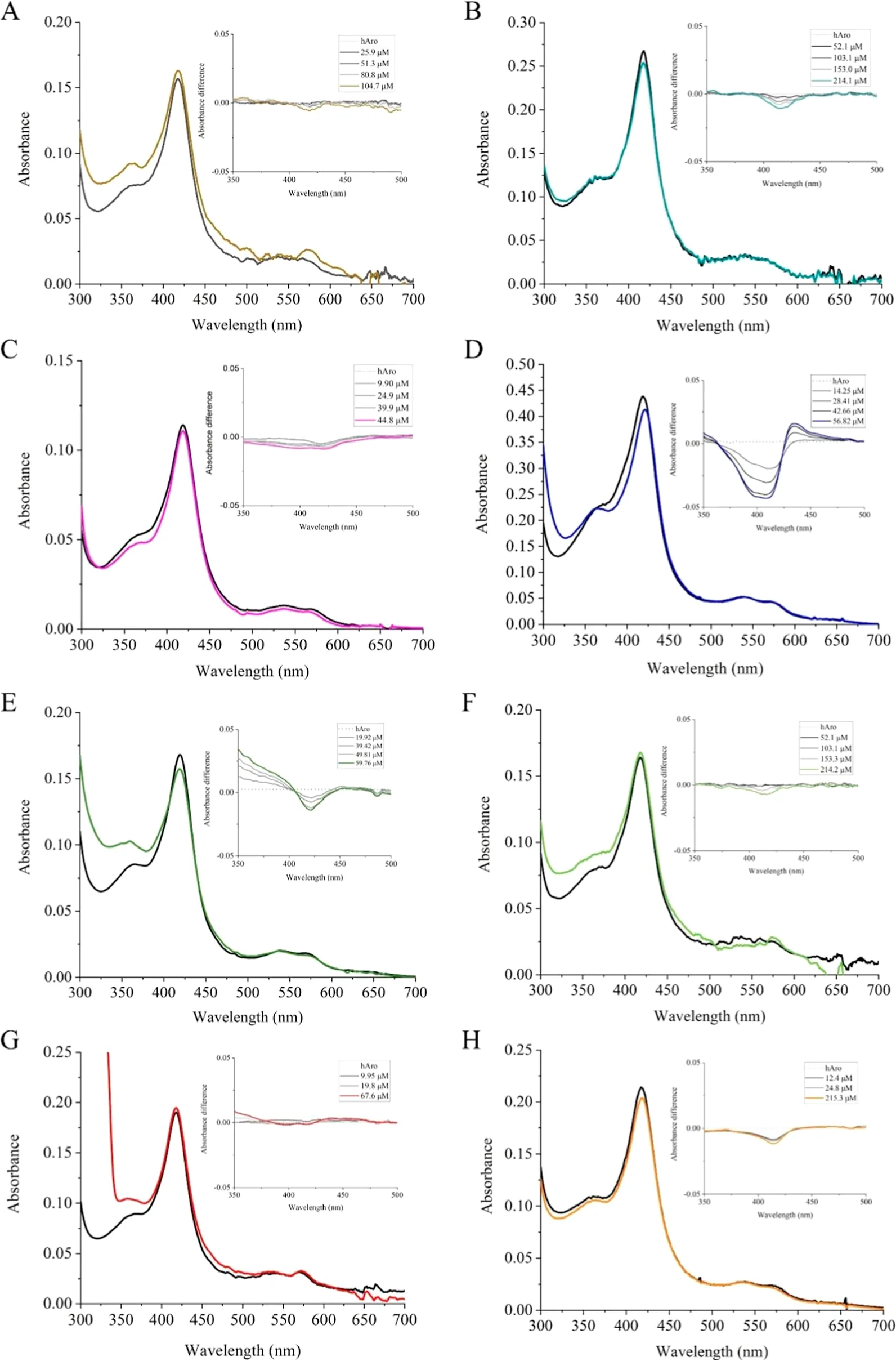
UV/vis absorption spectra of ligand-free aromatase (solid black line) and aromatase titrated (colored) with increasing concentrations of 2,6-dichlorobenzamide (A), AMPA (B), azoxystrobin (C), imazamox (D), metolachlor (E), nicosulfuron (F), quinclorac (G), and terbuthylazine-desethyl (H). Inset: difference spectra of pesticide-bound aromatase minus pesticide-free aromatase.

Instead, a type I shift was observed upon aromatase titration with metolachlor, showing a decrease in intensity at 418 nm and a slight increase at 394 nm, with an isosbestic point identified at 405 nm (Figure 1E). The intrinsic absorption band at 350 nm for metolachlor prevented *K*_D_ calculation. On the contrary, a type II spectroscopic shift was evident in the presence of imazamox, which is highlighted by the absorbance reduction at 418 nm and by the increase at 425 nm in the difference spectra (Figure 1D, inset), thus confirming the active site binding prediction of molecular docking simulations.

The spectral transitions (ΔAbs422 – ΔAbs418) obtained from aromatase titration with increasing concentrations of imazamox were plotted against the concentration of the pesticide, thus obtaining a hyperbolic curve (Figure 2). A dissociation constant (*K*_D_) of 26.3 ± 3.0 μM was computed by fitting the data with the previously mentioned equation (Materials and Methods 2.4). The calculated *K*_D_ is almost a hundred times that of the known aromatase inhibitor anastrozole (0.29 ± 0.03 μM),^27^ suggesting a weaker binding for imazamox.

**Figure 2:**
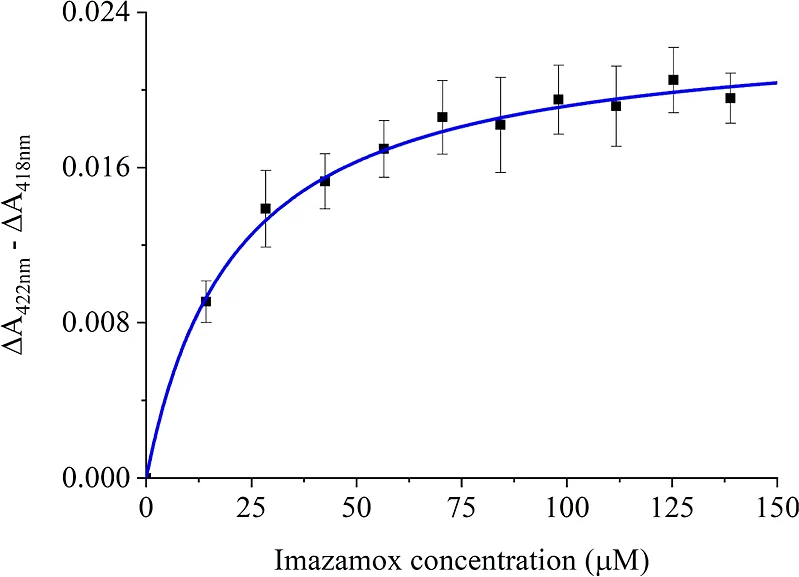
Binding curve resulting from aromatase titration with increasing amounts of imazamox, with a computed *K*_D_ of 26.3 ± 3.0 μM. *R*-Squared (hyperbolic model) = 0.988. Results are represented as means of three independent replicates and standard errors of the mean (*n* = 3).

The difference in the *K*_D_ value predicted by molecular docking and the one experimentally observed can be explained by the fact that docking simulations do not consider protein flexibility and do not predict direct heme coordination by the compound, which was observed by a type II spectral shift in the binding experiments. Therefore, the binding mode is predicted in the active site of the enzyme, but it is different from the real one. Moreover, a conformational change may also be induced by ligand binding and would affect not only the enthalpic term of the energy function but also the entropic term, which is difficult to predict by docking simulations.

### Effects of Selected Pesticides on Aromatase Activity

An activity assay was used to investigate the potential interference of the eight pesticides with the aromatase-mediated conversion of the substrate androstenedione (ASD) into product E1. An ASD concentration of 0.5 μM was chosen to maintain reaction conditions as close as possible to the enzyme *K*_M_ of 0.46 ± 0.06 μM.^36^ Reactions in the absence of any candidate inhibitor were used as positive controls (C+), in which ASD was totally consumed after 10 min of incubation.

The pesticides were first tested at concentrations of 10 and 50 μM, and the enzymatically produced E1 in their presence was quantified using HPLC. Control reactions were also performed in the presence of 1% and 2.5% of organic solvents without any pesticide, corresponding to the maximum concentration of solvent used during the assay (Figure S2). The results reported in Figure 3 show that 10 μM imazamox and nicosulfuron significantly reduce aromatase activity by 15.0 and 19.4%, respectively, while higher concentrations (50 μM) did not exert a significantly increased inhibitory effect. Consequently, imazamox and nicosulfuron could behave as aromatase partial inhibitors as they are not able to fully disrupt aromatase activity even in the presence of increased pesticides concentrations.

**Figure 3:**
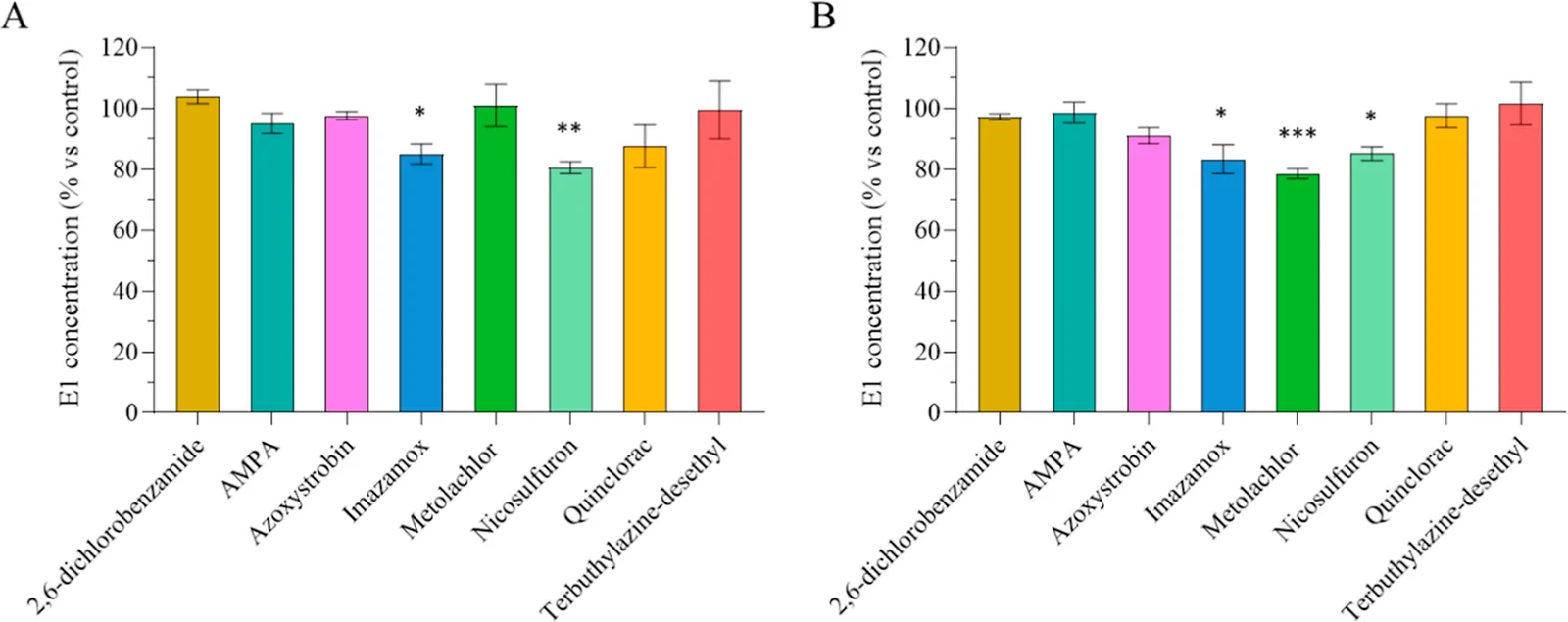
Mean estrone concentrations, expressed as percentages of E1 production in the control without any pesticide (100%), obtained from reactions in the presence of 10 (A) and 50 μM (B) of pesticides. All the reactions were incubated for 10 min at 30 °C. Results are represented as means of three independent replicates and standard errors of the mean (*n* = 3). * *p*-value <0.05 versus positive control (C+), *** *p*-value <0.001 vs C+, Shapiro–Wilk test followed by a two-tailed unpaired *t*-test with Welch’s correction.

Pesticides 2,6-dichlorobenzamide, AMPA, quinclorac, and terbuthylazine-desethyl did not inhibit aromatase activity either at 10 μM or at 50 μM, while metolachlor induced a significant inhibition of 21.4% only at 50 μM. To investigate whether higher metolachlor concentrations could enhance the observed inhibitory effect, the pesticide was also tested at 75 and 100 μM. The inhibition of aromatase activity increased to 23.7% and 27.2% in the presence of 75 and 100 μM metolachlor, respectively (Figure S3). However, this increase was not statistically significant if compared with the effect observed at 50 μM.

According to the docking simulations (Table 2) and to the UV–vis binding assay (Figure 1D–F), imazamox and metolachlor inhibit aromatase by binding in the active site, while nicosulfuron binds the enzyme in the allosteric site 1, proximal to the substrate access channel.^43^

A deeper investigation for imazamox and nicosulfuron was attempted to establish a dose–response curve by using a direct competitive ELISA specific for estradiol (E2), the most potent estrogen hormone. While nicosulfuron was found to interfere with the assay, for imazamox, it was possible to obtain a dose–response curve (Figure S4) and to calculate an IC50 of 5.2 ± 0.8 μM. This value is much higher than the contamination level detected in a water sample (Table 1), but little is known about its presence in environmental and biological matrices even if this pesticide is highly persistent in both soil and water (Table S1).

### Thermodynamics of Pesticide Binding to Aromatase by ITC

The interaction of imazamox and metolachlor with aromatase was studied from a thermodynamic point of view by isothermal titration calorimetry. Such an analysis was not possible for nicosulfuron due to the poor solubility in the buffer used for the titration.

Imazamox was titrated in the aromatase solution prepared in a 100 mM KPi (pH 7.4), 0.1% Tween 20, and 2.5% glycerol buffer up to a molar ratio of 9:1 (pesticide/aromatase) (Figure 4A). The obtained signals were integrated and fitted using the equation indicated in the Materials and Methods section. The fitting of the data allowed the calculation of the stoichiometry and the thermodynamic parameters of binding of imazamox to aromatase (Figure 4B). Imazamox binds exothermically to aromatase in a 1:1 stoichiometry, with a significant entropic contribution and a dissociation constant (*K*_D_) of 18.76 ± 0.82 μM, which is in line with the one calculated from UV/vis binding assay (26.3 ± 3.0 μM).

**Figure 4:**
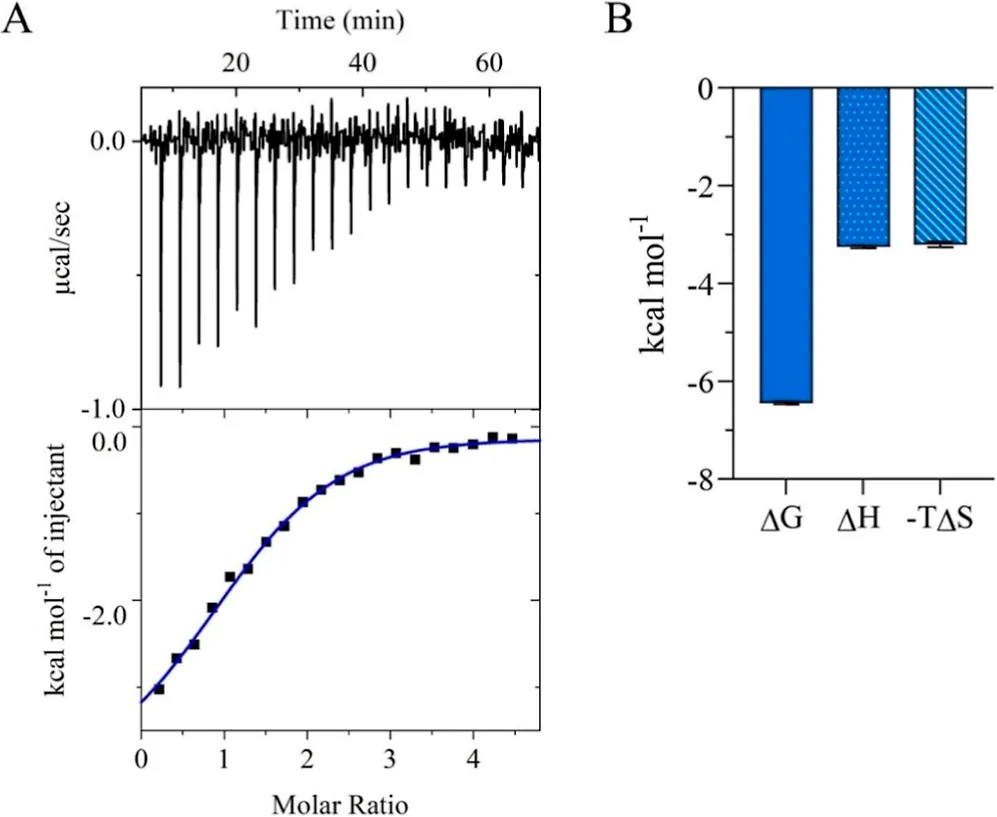
ITC results for the analysis of imazamox binding to aromatase. (A) Injections heat signals (top) and fitted data (enthalpy changes vs molar ratio, bottom) (B) Thermodynamic parameters of aromatase binding to imazamox computed from ITC data analysis. Error bars: 95% confidence interval.

The titration of aromatase was repeated using the same experimental conditions with metolachlor as the ligand up to a molar ratio of 3:1 (pesticide/aromatase). No interaction between aromatase and the pesticide was detected (data not shown). The analysis was then carried out in a 100 mM KPi (pH 7.4), 0.1% Tween-20 buffer solution containing 10% glycerol. A weak endothermic binding was detected (Figure 5A), resulting in a *K*_D_ of 1.77 ± 0.74 μM, similar to the one predicted by molecular docking simulations. The thermodynamic parameters (Figure 5B) showed an even higher entropic contribution in the binding of metolachlor to aromatase. It was hypothesized that the presence of a higher percentage of glycerol in the buffer could slow down the dynamic fluctuations of aromatase structure, thus favoring the selection of a conformation more prone to interact with metolachlor. Indeed, as previously reported, the aromatase substrate binding mechanism follows a conformational selection mode preferably over an induced fit one.^39^ This means that the substrate binds one of the possible conformations that the enzyme dynamically samples. On the contrary, an induced fit mechanism implicates a ligand-induced conformational rearrangement that optimizes the substrate–enzyme interaction.^48^ As previously observed for substrate binding in aromatase, the significant entropic contribution observed suggests that pesticide binding may involve conformational selection and desolvation effects, supporting a dynamic conformational selection model of aromatase–pesticide interaction rather than a more rigid induced fit mechanism.

**Figure 5:**
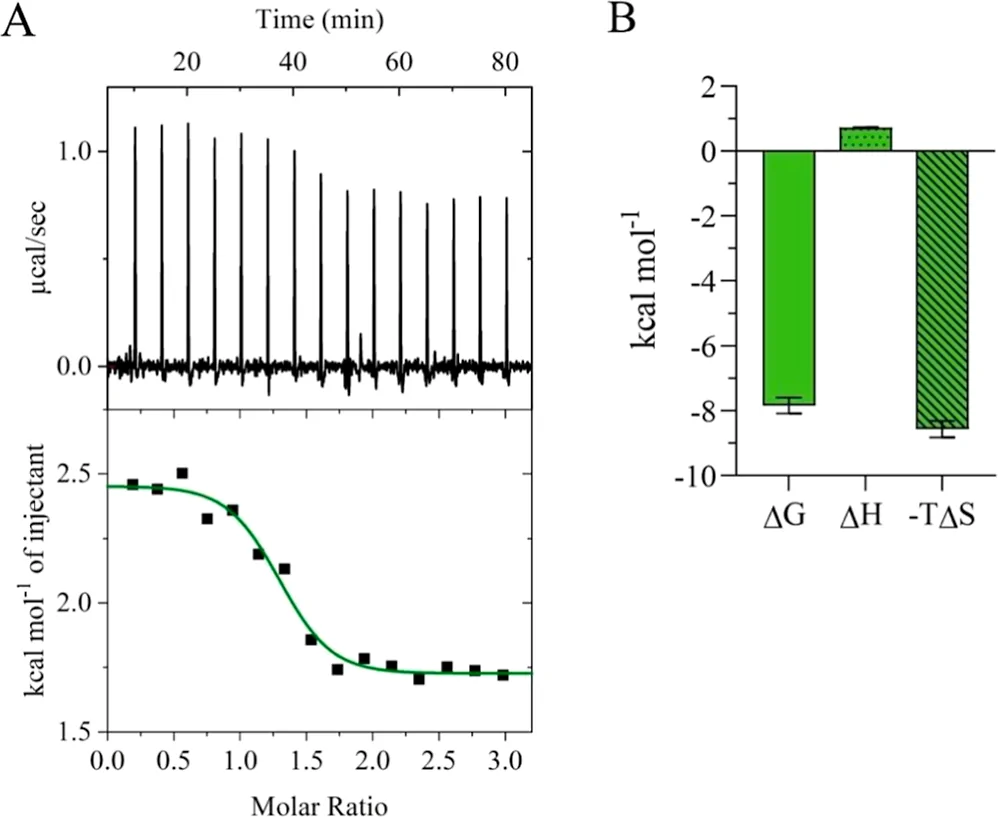
ITC for the analysis of metolachlor binding to aromatase. (A) Injections heat signals (top) and fitted data (enthalpy changes vs molar ratio, bottom). (B) Thermodynamic parameters of aromatase binding to metolachlor computed from ITC data analysis. Error bars: 95% confidence interval.

### Pesticides Estrogenic Activity Assessment

An MELN gene reporter assay was carried out to define a possible interference of the eight investigated pesticides with the nuclear ER signaling.

AMPA, 2,6-dichlorobenzamide, azoxystrobin, imazamox, metolachlor, and nicosulfuron cannot be considered nuclear ER agonists as no significant estrogenic activity is observed in the presence of these pesticides (Figure 6A–F). On the contrary, increasing concentrations of quinclorac and terbuthylazine-desethyl show an increase in relative luciferase activity signal, statistically significant at concentrations between 6.25 × 10^–5^ and 1.00 × 10^–3^ M for quinclorac (from −4.2 to −3.0 log­(concentration M)) (Figure 6G) and from 3.91 × 10^–6^ to 1.00 × 10^–3^ M for terbuthylazine-desethyl (from −5.4 to −3.0 log­(concentration M)) (Figure 6H). These concentrations are considerably higher than those of E2 inducing the major estrogenic activity (10^–10^ to 10^–8^ M, data not shown) and of those found in waters.

**Figure 6:**
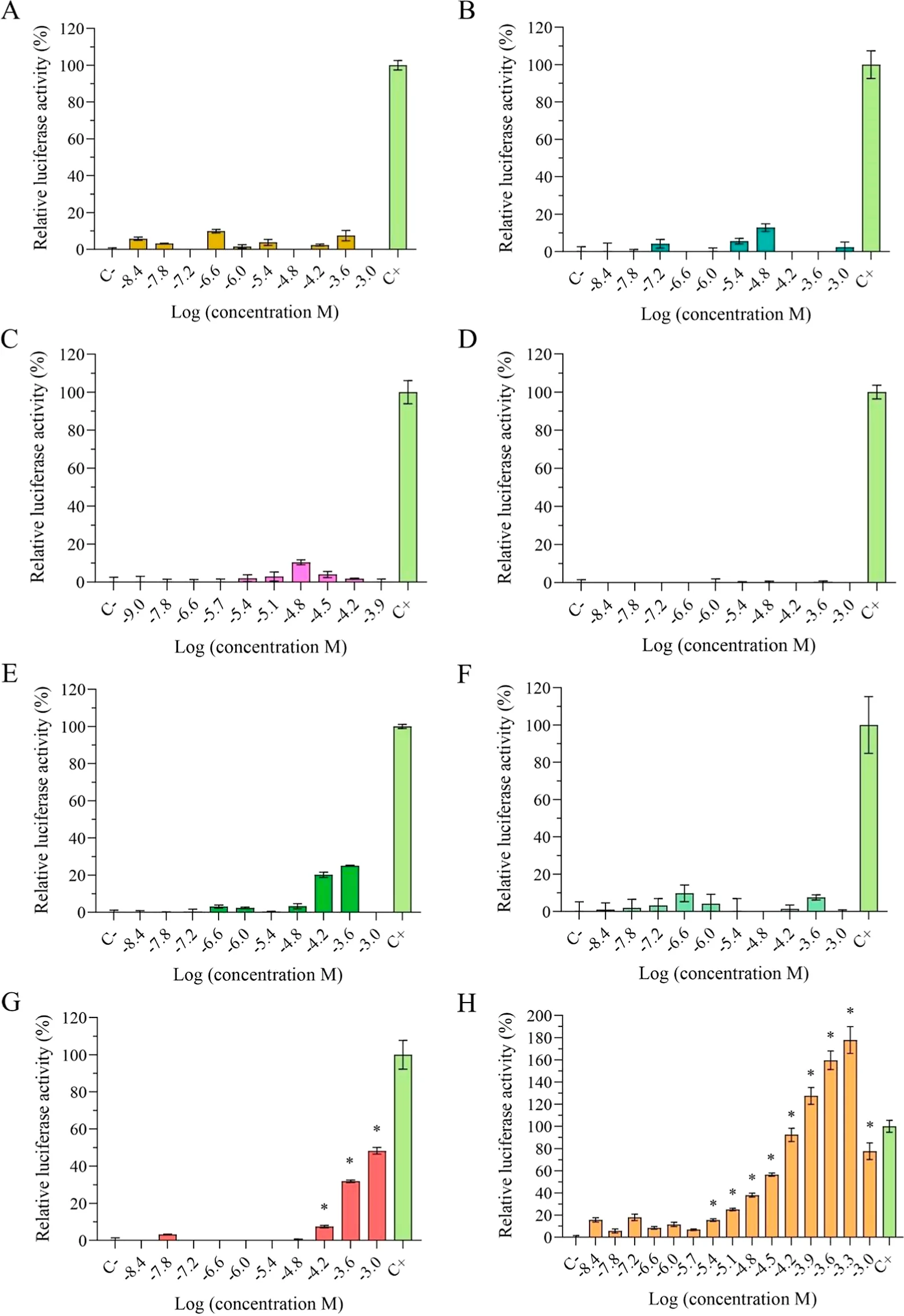
Estrogenic activity of 2,6-dichlorobenzamide (A), AMPA (B), azoxystrobin (C), imazamox (D), metolachlor (E), nicosulfuron (F), quinclorac (G), and terbuthylazine-desethyl (H). Data are expressed as relative luciferase activity (C+ = 10^–8^ M E2, luciferase activity = 100%) and represented as means and standard deviations of two independent experiments (*n* = 2), each performed in quadruplicate. *p-Value < 0.05, Kruskal–Wallis test followed by Dunnett’s post hoc test.

Quinclorac and terbuthylazine-desethyl estrogenic activities were expressed in terms of estrogenic potency by calculating the Effective Concentration 50 (EC50) and the E2 Equivalency Factor (EEF). Quinclorac and terbuthylazine-desethyl show a mean EC50 of 9.02 × 10^–4^ (95% confidence interval: 7.44 × 10^–4^ to 1.14 × 10^–3^) and 1.83 × 10^–5^ M (95% confidence interval: 1.58 × 10^–5^ to 2.14 × 10^–5^), respectively, which are considerably higher than that of E2 (7.72 × 10^–12^ M). Consequently, the EEF values of both the pesticides are significantly low: 8.55 × 10^–9^ (95% confidence interval: 6.79 × 10^–9^ to 1.04 × 10^–8^) for quinclorac and 4.22 × 10^–7^ (95% confidence interval: 3.60 × 10^–7^ to 4.89 × 10^–7^) for terbuthylazine-desethyl, which presents the highest potency.

Then, 1.00 × 10^–3^ M quinclorac (−3.0 log­(concentration M)) and 3.13 × 10^–5^ M terbuthylazine-desethyl (−4.2 log­(concentration M)) were tested in combination with 10^–11^ M E2 or 10^–6^ M *T* as controls of ERs involvement in mediating the observed effects (Figure S4). The increase in luciferase activity upon cell coexposure to quinclorac or terbuthylazine-desethyl and E2 suggests a possible synergistic activity. Moreover, the presence of tamoxifen reduces to 0% the detected signal, providing evidence that the observed effects depend on nuclear ERs since tamoxifen competes with E2 for receptor binding. The possible antiestrogenic activity of the pesticides resulting in negative ER agonism was investigated by coexposing the cells to 3.91 × 10^–6^ M and 6.25 × 10^–5^ M (−5.4 and −4.2 log­(concentration M)) pesticides (2.44 × 10^–7^ M and 3.91 × 10^–6^ M for azoxystrobin) and 10^–11^ M E2 (Figure 7). None of them significantly affect the relative luciferase activity, which remains comparable to that obtained with the reference treatment (10^–11^ M E2 alone).

**Figure 7:**
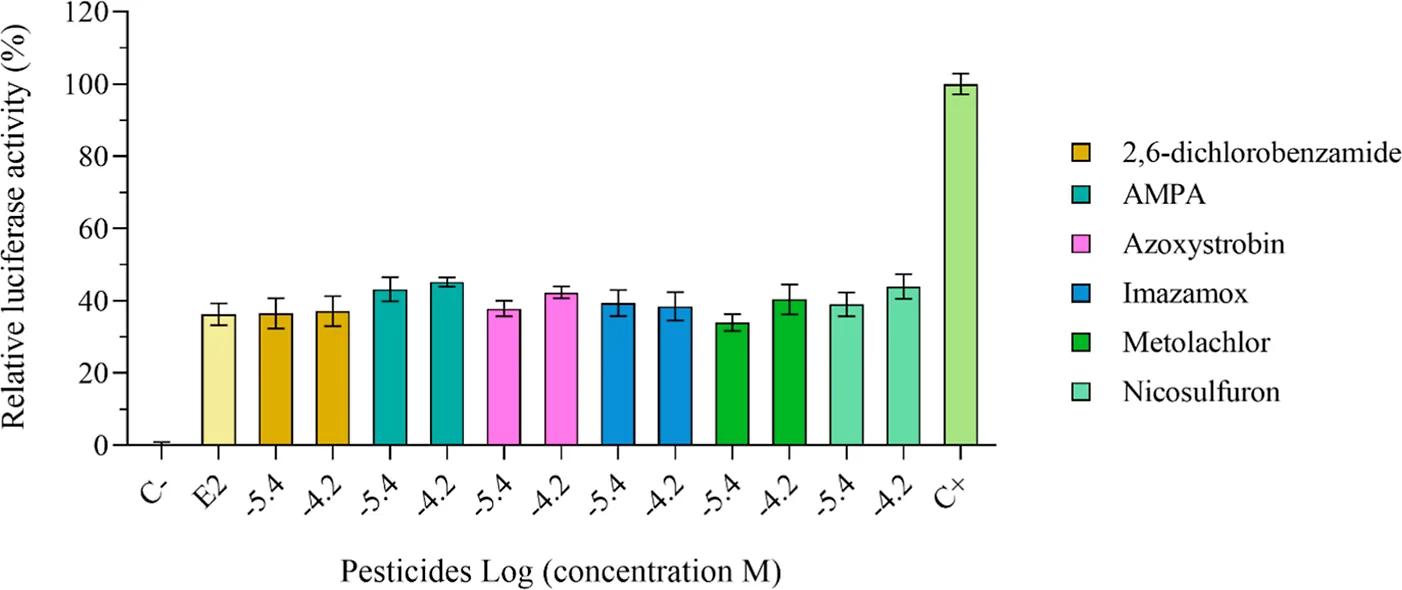
Estrogenic activity of 2,6-dichlorobenzamide (dichlo), AMPA, azoxystrobin (azo), imazamox (ima), metolachor (met), and nicosulfuron (nico) in the presence 10^–11^ M E2. Data are expressed as relative luciferase activity (C+ = 10^–8^ M E2, luciferase activity = 100%) and represented as means and standard deviations of two independent experiments (*n* = 2), each performed in quadruplicate.

The EEFs of 8.55 × 10^–9^ (quinclorac) and 4.22 × 10^–7^ (terbuthylazine-desethyl) indicate that high concentrations of pesticides are required to achieve the same effect of the endogenous ligand E2. The obtained EEFs are in line with those of other previously tested pesticides, such as thiacloprid (EEF: 3.7 × 10^–9^).^42^ The same assay used for the study of other emerging pollutants, such as halophenolic disinfection byproducts, showed comparable EEF values, e.g., 4.41 × 10^–8^ for 2,4,6-triiodophenol.^49^ In addition, as a comparison with well-known EDCs, bisphenol A showed an EC50 of 1.25 × 10^–6^ M on the same cell line,^50^ close to that of terbuthylazine-desethyl, and an EEF value of 1.8 × 10^–5^.^51^

## Discussion

This study provides evidence of commonly used pesticides as new potential EDCs that interfere with estrogen synthesis and/or signaling.

Eight pesticides, detected in the ground and surface water of Piedmont, Italy, were selected to be screened on aromatase and ERα. Imazamox, metolachlor, and nicosulfuron were found to partially inhibit aromatase activity as they cannot fully disrupt aromatase activity even at the highest concentrations tested. The partial effect could be due to an allosteric inhibitory modulation or to a competitive mechanism that does not completely avoid the entry of the substrate into the active site of the protein and/or it is too weak to be fully competitive with the substrate. The last hypothesis is supported by the calculated *K*_D_ values for imazamox and metolachlor of 18.8 ± 0.8 and 1.8 ± 0.7 μM, respectively. As for reference, the measured K_D_ value for aromatase substrate androstenedione is 1.2 ± 0.1 μM and the one for anastrozole, which is a complete aromatase inhibitor, is 0.29 ± 0.03 μM, which is consistent with an almost full depletion of aromatase activity, leaving less than 5% of residual activity.^27^

The first two act by directly interacting with the enzymatic active site, while the third (most probably) by allosteric modulation. Low micromolar dissociation constants characterized imazamox and metolachlor binding to aromatase (18.8 ± 0.8 and 1.8 ± 0.7 μM, respectively), while an IC50 of 5.2 ± 0.8 μM for imazamox was obtained from competitive ELISA assays. Moreover, MELN gene reporter assay revealed that quinclorac and terbuthylazine-desethyl induced nuclear ERα-mediated transcriptional activation with a mean EC50 of 9.02 × 10^–4^ and 1.83 × 10^–5^ M, respectively.

However, a critical evaluation of the pesticides’ endocrine disruptive potential must consider the actual human exposure to these chemicals. The highest concentrations found in surface and ground waters range in the nM to low μM order of magnitude (0.006–2.10 μM),^9,10^ which would be further diluted in the whole human body system. According to the results of this work, the IC50 value calculated for imazamox is 2 orders of magnitude higher than the concentration found in environmental matrices. However, no data on human exposure are available. On the contrary, for metolachlor, the *K*_D_ value calculated is only 3 folds higher than the concentration reported in the literature in urinary samples.^19^ As for the pesticides found to be ER agonists, the EC50 values are much higher than the concentrations detected and reported in the previous work (Table 1) even if the value for terbuthylazine-desethyl is not so far from the one reported for bisphenol A, a well-known EDC with weak estrogenic activity. Moreover, the lack of bioaccumulation data for all these chemicals makes it difficult to determine the actual concentrations to which endocrine targets are exposed, which can be higher than those found in the environment. In addition, pesticides are frequently used in agriculture as mixtures of different single active compounds among which toxicokinetic and toxicodynamic interactions can occur, resulting in potentiated, additive, and synergic effects,^52^ as demonstrated for metolachlor by Dhanwada et al. (2003).^53^

The recent literature revealed that imazamox is highly persistent in soil (over 90 days),^54^ and as of now, it does not meet the European Union requirements to be identified as an EDC, lacking a comprehensive understanding of human exposure to the pesticide and of its carcinogenic and genotoxic potentials.^55^ On the contrary, both imazamox and nicosulfuron meet the criteria to be considered persistent and toxic; therefore, in Europe, they have been inserted in the list of pesticides candidate for replacement.^56^ The assessment of metolachlor endocrine-disrupting activity is critical in several areas of concern.^57^ It could constitute a good EDC candidate as it can affect the mammalian reproductive system and can enhance the probability of developing leukemia, lymphoma, and bone cancers.^21,58^

Terbuthylazine, the terbuthylazine-desethyl parental compound, has been already defined as a carcinogen category 3,^59^ with a proven ability to alter steroidogenesis^60^ and a potential estrogenic activity with lower persistence and lower bioactivity than its metabolite.^61^ Furthermore, there is a significant lack of data related to possible effects of quinclorac on human health. It seems to have low toxicity on mammals but negatively affects microbial populations of the soil in terms of their abundance and composition.^62^

As endocrine regulation is a complex system that involves different pathways of hormone synthesis, signaling, and metabolism, the presented results should be integrated with further *in vitro* and *in vivo* studies, which should also consider other endocrine targets, in order to achieve a full understanding of biologically active concentrations and mechanism of action of these pesticides.

## Supplementary Material



## Notes

Tested pesticides with persistence data and literature reports about their effect on the estrogen-related endocrine system (Table S1); chemical properties and composition of the relative stock solutions of pesticides (Table S2); description of the HPLC acetonitrile linear gradient (Table S3); UV/vis spectra of aromatase titration with solvents (Figure S1), control of estrone production by aromatase in the presence of solvents (Figure S2), estrone production by aromatase in the presence of 50, 75, and 100 μM metolachlor (Figure S3), dose–response curve for aromatase activity in the presence of imazamox obtained from E2 quantification by ELISA assay (Figure S4), and estrogenic activity of quinclorac and terbuthylazine-desethyl in the presence of 10^–11^ M E2 or 10^–6^ M tamoxifen (Figure S4) (PDF)
