# Supplementary material for: Environmentally Persistent Pesticides: In Vitro Effects on Human Aromatase Activity and ERα Signaling
Source: Chem Res Toxicol. 2026 Aug 13;39(9):1723–34. doi: 10.1021/acs.chemrestox.6c00151 (PMC13599381; doi:10.1021/acs.chemrestox.6c00151)
Supplement: Supplementary file 1 [file tx6c00151_si_001.pdf]

Supporting information

Environmental persistent pesticides: *in vitro* effects on human aromatase activity and ERα signalling

Francesca Armellino,[†] Sabrina Costa,[§] Francesco Giaretto,[†] Michela Rossi,[†] Gianluca Catucci,[†] Gianfranco Gilardi,[†] Tiziana Schilirò,[§,] Giovanna Di Nardo[†,*]*

[†] Department of Life Sciences and Systems Biology, University of Torino, 10123 Torino, Italy

[§] Department of Sciences of Public Health and Pediatrics, University of Torino, 10126 Torino, Italy

* Corresponding authors: Giovanna Di Nardo: giovanna.dinardo@unito.it, Tiziana Schilirò: tiziana.schiliro@unito.it.

CONTENTS:

Supporting Tables: tested pesticides with persistence data and literature reports about their effect on estrogen-related endocrine system (Table S1); chemical properties and composition of the relative stock solutions of pesticides (Table S2); description of HPLC acetonitrile linear gradient (Table S3). **Supporting Figures**: UV/Vis spectra of hAro titration with solvents (Figure S1), control of estrone production by hAro in presence of solvents (Figure S2), estrone production by aromatase in presence of 50, 75 and 100 μM metolachlor (Figure S3), dose-

response curve for hAro activity in presence of imazamox obtained from E2 quantification by ELISA assay (Figure S4), estrogenic activity of quinclorac and terbuthylazine-desethyl in presence of 10^{-11} M E2 or 10^{-6} M tamoxifen (Figure S5).

Table S1. Data on persistence of pesticides expressed as their reported half-life (DT50, expressed in days) from PPDB[1] and reported estrogen-disrupting effect.

	**DT50 soil (typical)**	**DT50 water sediment**	**DT50 water phase**	**Reported estrogen-disrupting effect**	**Reference**
**2,6-dichlorobenzamide**	137.7	10000	10000	NO	
**AMPA**	114	132	5.47	YES	Tóth *et al.*, 2024[2]
**Azoxystrobin**	78	205	6.1	YES	Halawa *et al.,* 2021[3]
**Imazamox**	200.2	233	132	NO	
**Metolachlor**	90	365	88	YES	Ou Yang *et al.,* 2022[4]
**Nicosulfuron**	29.5	60.6	83.8	NO	
**Quinclorac**	450		21[a]	NO	
**Terbuthylazine-desethyl**	54		102-366[b]	YES	Pereira et al., 2015[7]

[a] Data from Dos Santos et al., 2005[5]

[b] Data from Navarro et al., 2004[6]

Table S2. Tested pesticides and solvents in which stock solutions were prepared. All data are available on PubChem database (https://pubchem.ncbi.nlm.nih.gov/). For Buffer A composition refer to *Materials and methods 2.3*. PBS: Phosphate Buffered Saline.

Name (CAS)	Purity	Molecular Weight (g/mol)	Molecular formula	Bioconcentration Factor (BCF)	Solvent
**2,6-dichlorobenzamide (2008-58-4)**	100.0%	190.02	$C_7H_5Cl_2NO$	<10	Kpi 0.1 M[a]<br>DMSO[b]
**AMPA (1066-51-9)**	99.0%	111.04	CH_6NO_3P	0.52 (glyphosate)	Kpi 0.1 M[a]<br>PBS[b]
**Azoxystrobin (131860-33-8)**	99.3%	403.4	$C_{22}H_{17}N_3O_5$	21	Acetonitrile[a]<br>DMSO[b]
**Imazamox (114311-32-9)**	99.7%	305.33	$C_{15}H_{19}N_3O_4$	3.2	Kpi 0.1 M[a]<br>DMSO[b]
**Metolachlor (51218-45-2)**	98.4%	283.79	$C_{15}H_{22}ClNO_2$	<1 and <69	Buffer A[a]<br>DMSO[b]
**Nicosulfuron (111991-09-4)**	99.4%	410.4	$C_{15}H_{18}N_6O_6S$	3	Kpi 0.1 M[a]<br>DMSO[b]
**Quinclorac (84087-01-4)**	≥ 99.9%	242.05	$C_{10}H_5Cl_2NO_2$	<1	Absolute ethanol[a]<br>DMSO[b]
**Terbuthylazine-desethyl (30125-63-4)**	99.6%	201.66	$C_7H_{12}ClN_5$	N.A	40% absolute ethanol (UV/Vis)[a]<br>Acetonitrile (HPLC)[a]<br>DMSO[b]

[a] Solvent for stock solutions used for activity and binding assays and ITC analysis. [b] Solvent for stock solutions for MELN gene reporter assay.

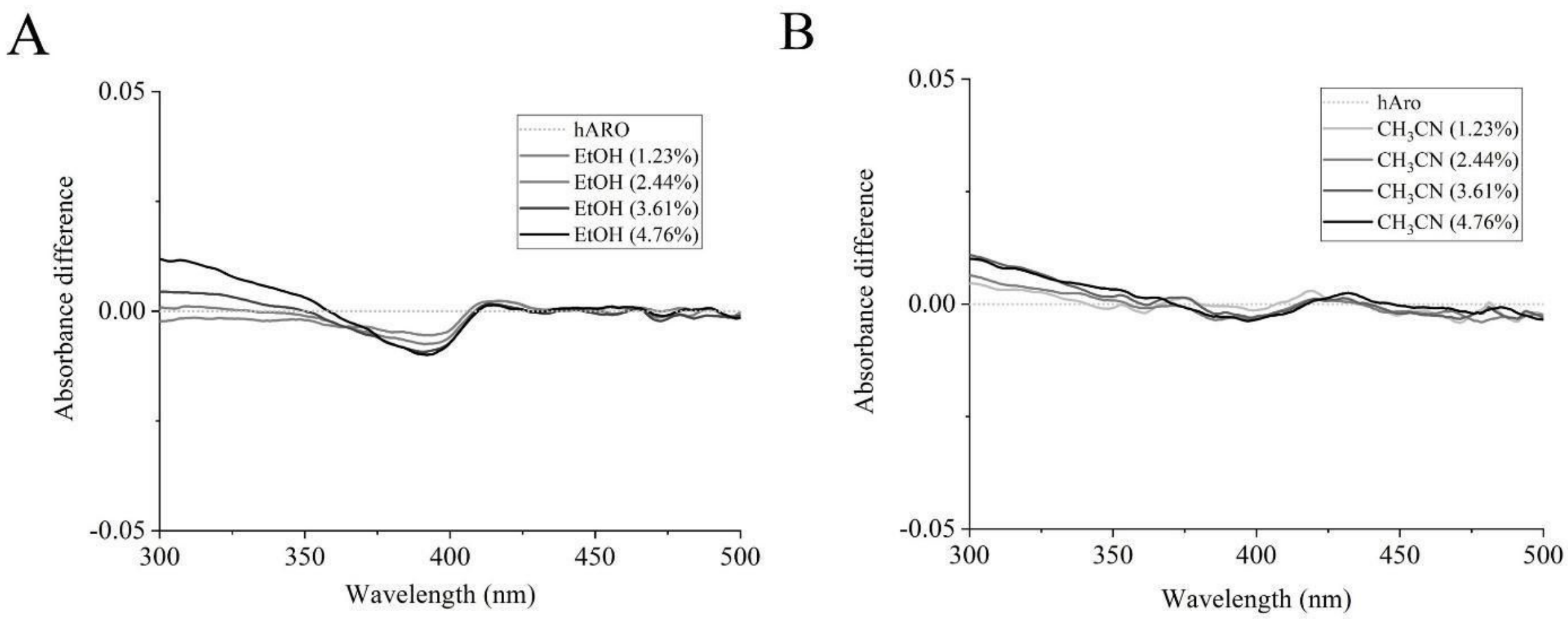

Figure S1. Difference UV/Vis spectra of hAro with absolute ethanol (A) or acetonitrile (B) minus hAro without solvent (black dotted line), obtained from hAro titration with increasing concentrations of solvent.

Table S3. Detailed HPLC-grade acetonitrile linear gradient mixed with milli-Q water.

Time (min)	MilliQ water (%)	Acetonitrile (%)
0	75	25
10	75	25
25	30	70
28	30	70
30	5	95
40	5	95

43	75	25
49	75	25

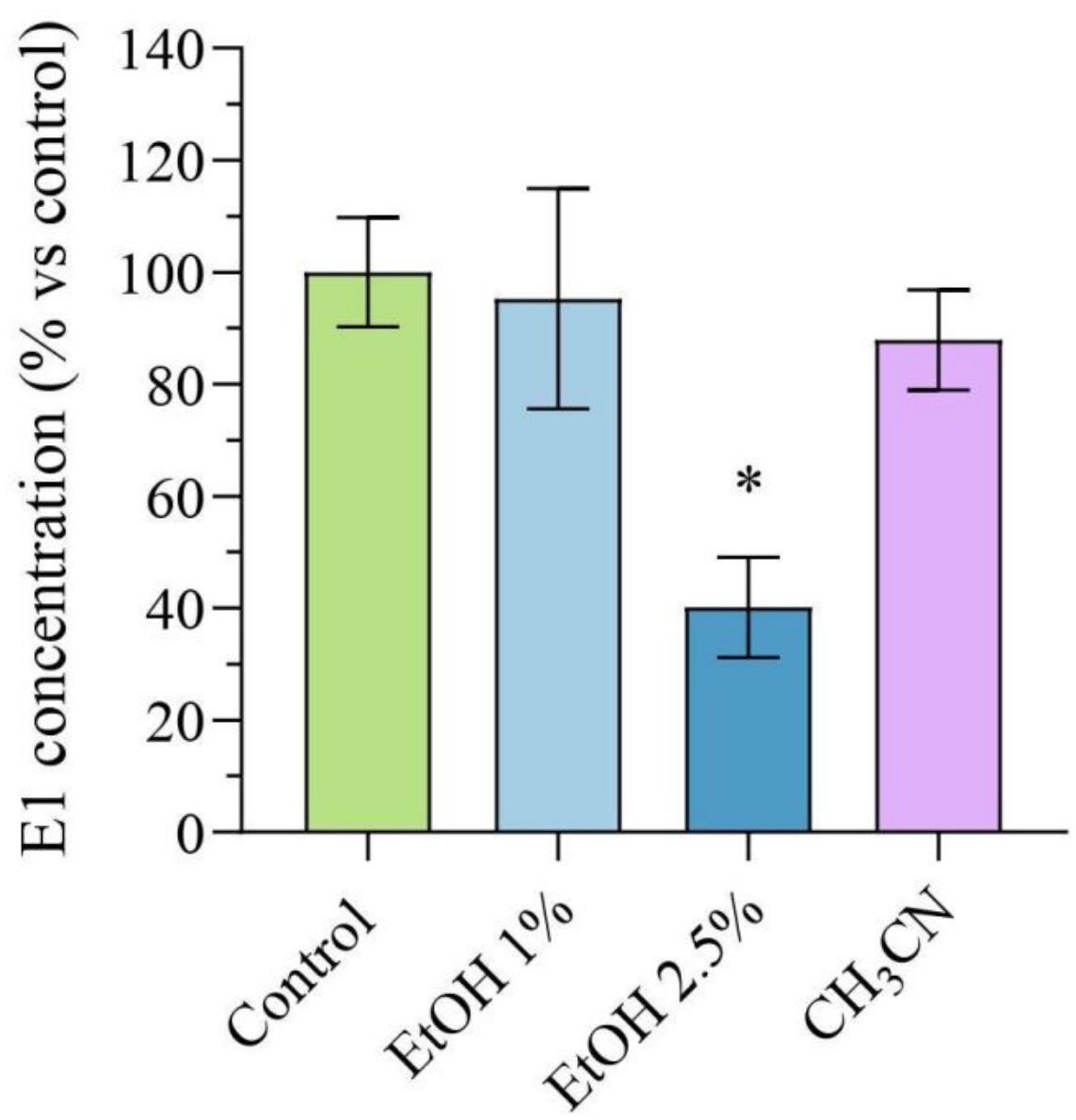

Figure S2. Mean estrone concentrations obtained from reactions in presence of absolute ethanol (EtOH) 1% and 2.5%, or CH_3CN 1%, expressed in percentage as relative estrone production (Control = 100% ASD conversion in E1 in absence of solvents). All the reactions were incubated for 10 min at 30 ˚C. Results are represented as means and standard errors of the mean (n=3).

* p-value <0.05 versus positive control (C+), Shapiro-Wilk test followed by a two-tailed unpaired t-test with Welch’s correction.

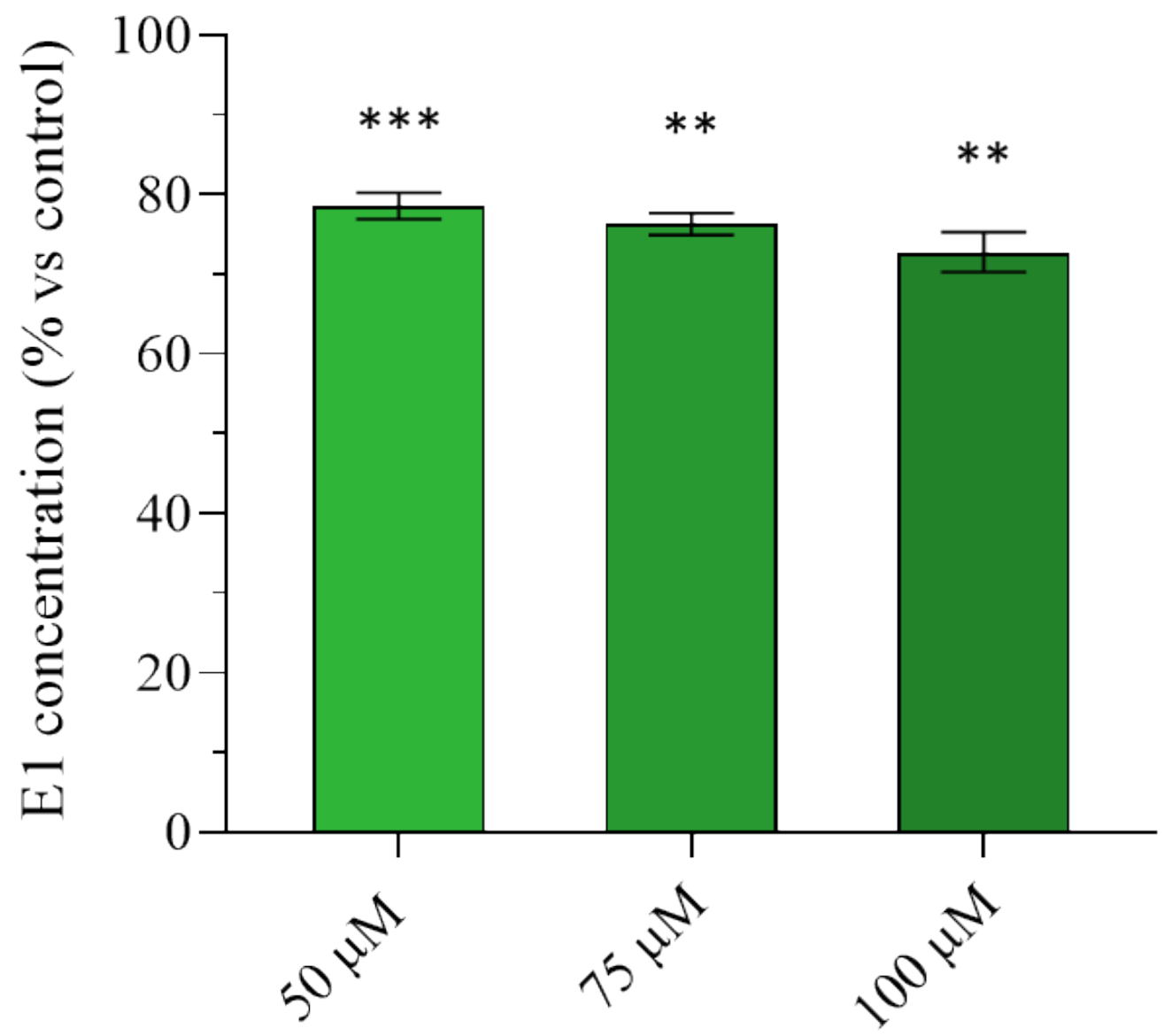

Figure S3. Mean estrone concentrations obtained from reactions in presence of 50, 75 and 100 µM metolachlor. All the reactions were incubated for 10 min at 30 ˚C. Results are represented as means and standard errors of the mean (n=3).

** p-value <0.01 versus positive control (C+), *** p-value <0.001 vs C+, Shapiro-Wilk test followed by a two-tailed unpaired t-test with Welch's correction.

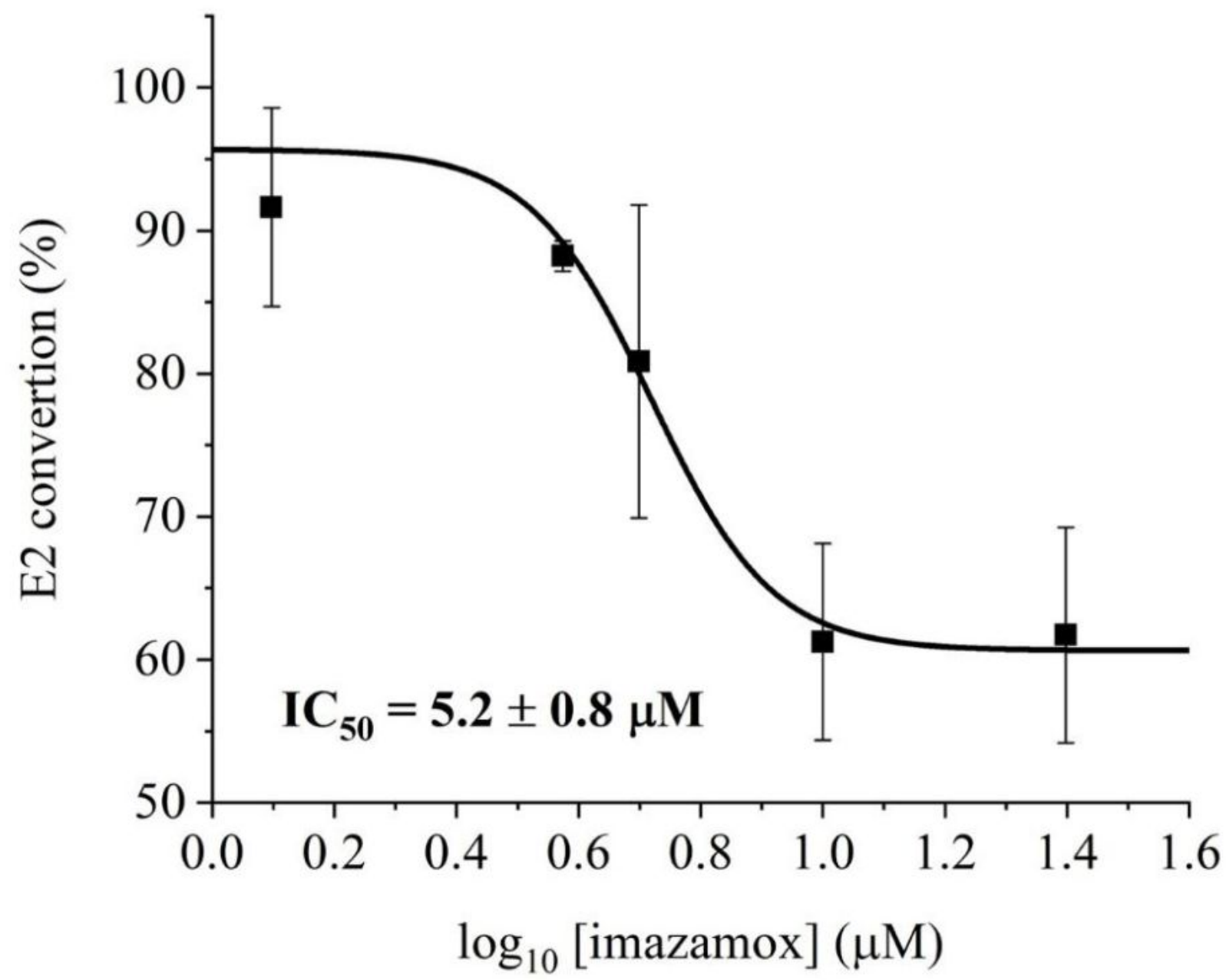

Figure S4. Dose-response curve for hAro activity in presence of imazamox obtained from E2 quantification by ELISA assay. Results are represented as means of three independent replicates and standard errors of the mean (n=3). Data were fitted according to the equation indicated in mentioned Materials and Methods section 2.5, allowing the calculation of an IC_{50} of 5.2±0.8 μM.

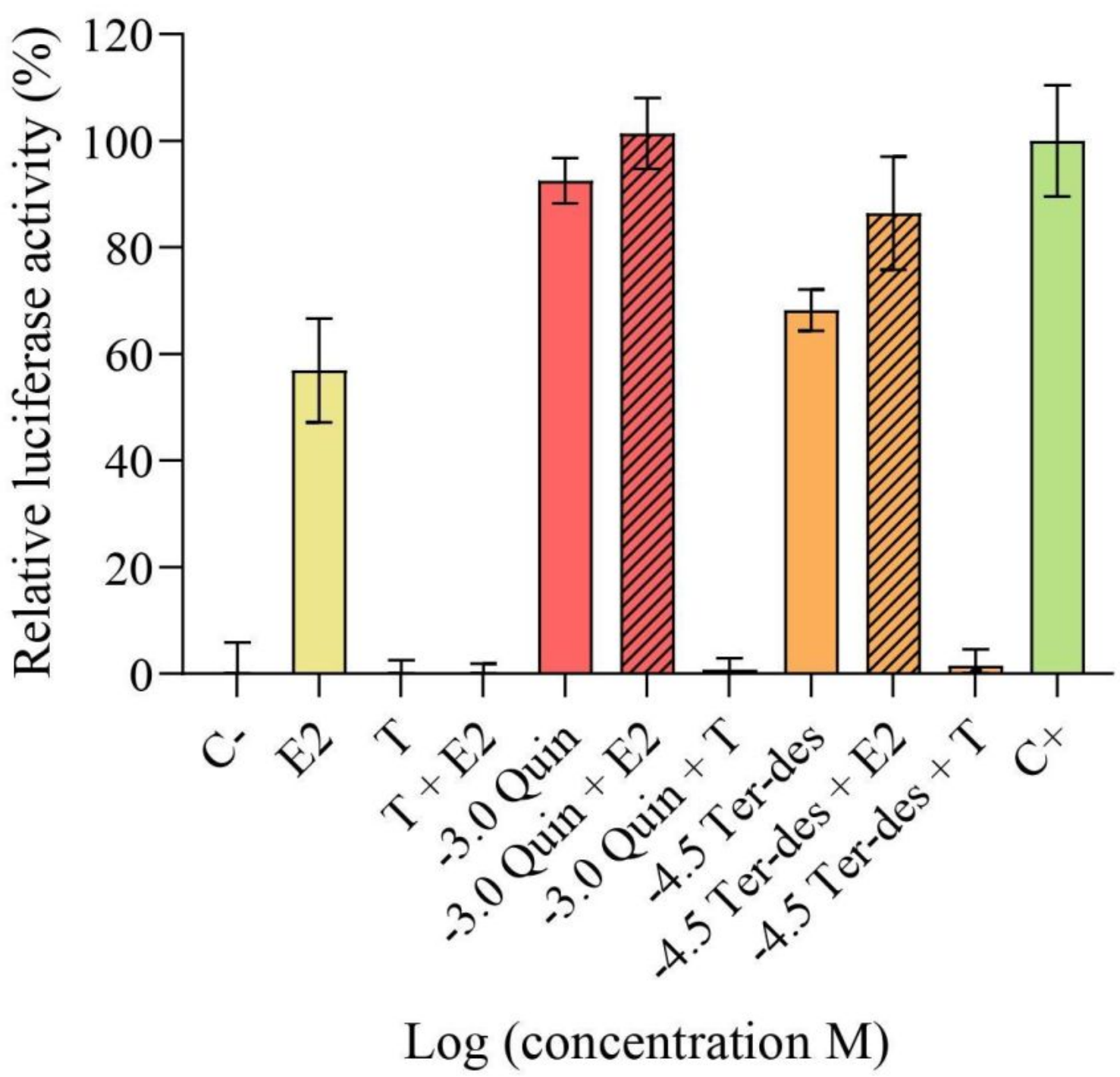

Figure S5. Estrogenic activity of quinclorac (quin) and terbuthylazine-desethyl (ter-des) in presence of 10^{-11} M E2 or 10^{-6} M tamoxifen (T), expressed as relative luciferase activity (C+ = 10^{-8} M E2, luciferase activity = 100%). Results are represented as means and standard deviations of two independent experiments (n=2), each performed in quadruplicate.

REFERENCES

(1) Lewis, K., Tzilivakis, J., Green, A., & Warner, D. (2006). Pesticide Properties DataBase (PPDB). Agriculture & Environment Research Unit, University of Hertfordshire. http://www.herts.ac.uk/aeru/ppd

(2) Tóth, G., Háhn, J., Szabó, G., Bakos, K., Volner, C., Liang, X., Göbölös, B., Bock, I., Szoboszlay, S., Urbányi, B., Kriszt, B., Kaszab, E., Szabó, I., Csenki, Z., 2024. In vivo estrogenicity of glyphosate, its formulations, and AMPA on transgenic zebrafish (Danio rerio) embryos. Environ. Pollut. 342, 123113. https://doi.org/10.1016/j.envpol.2023.123113

(3) Halawa, E., Ryad, L., El-Shenawy, N.S., Al-Eisa, R.A., EL-Hak, H.N.G., 2021. Evaluation of acetamiprid and azoxystrobin residues and their hormonal disrupting effects on male rats using liquid chromatography-tandem mass spectrometry. PLoS ONE 16, e0259383. https://doi.org/10.1371/journal.pone.0259383

(4) Ou Yang, K., Feng, T., Han, Y., Li, G., Li, J., Ma, H., 2022. Bioaccumulation, metabolism and endocrine-reproductive effects of metolachlor and its S-enantiomer in adult zebrafish (Danio rerio). **Sci. Total Environ.** 802, 149826. https://doi.org/10.1016/j.scitotenv.2021.149826

(5) Dos Santos Miron, D., Crestani, M., Rosa Shettinger, M., Maria Morsch, V., Baldisserotto, B., Angel Tierno, M., Moraes, G., Vieira, V.L.P., 2005. Effects of the herbicides clomazone, quinclorac, and metsulfuron methyl on acetylcholinesterase activity in the silver catfish

(Rhamdia quelen) (Heptapteridae). Ecotoxicol. Environ. Saf. 61, 398–403. https://doi.org/10.1016/j.ecoenv.2004.12.019

(6) Navarro, S., Vela, N., José Giménez, M., Navarro, G., 2004. Persistence of four s-triazine herbicides in river, sea and groundwater samples exposed to sunlight and darkness under laboratory conditions. **Sci. Total Environ.** 329, 87–97. https://doi.org/10.1016/j.scitotenv.2004.03.013

(7) Pereira, S. V., Reis, T., Souza, B. S., Dantas, R. F., Azevedo, D. A., Dezotti, M., ... Esplugas, S., 2015. Oestrogenicity assessment of s-triazines by-products during ozonation. Environ. Technol. 36(12), 1538-1546. https://doi.org/10.1080/09593330.2014.995235
